# Water Status and Flavor Evolution in Vacuum Freeze-Dried Mulberry: A Potential Flavor Transition Point

**DOI:** 10.3390/foods15152618

**Published:** 2026-07-27

**Authors:** Shuang Bi, Mengjia Ren, Chunhe Shi, Fan Yang, Xin Pan, Jihong Wu, Ye Liu

**Affiliations:** 1 Flavor Science Laboratory, School of Food and Health, Beijing Technology and Business University (BTBU), Beijing 100048, China; 2 School of Perfume and Aroma Technology, Shanghai Institute of Technology, Shanghai 201418, China; 3 College of Food Science and Nutritional Engineering, China Agricultural University, Beijing 100083, China

**Keywords:** mulberry, vacuum freeze drying, water status, key aroma-active compounds, flavor turning point

## Abstract

Vacuum freeze drying significantly influences the water status and aroma characteristics of mulberry. This study monitored the dynamic changes in water distribution, non-volatile components, and key aroma-active compounds during a 72 h drying process. The results showed that free water decreased from 85.3% to 5.96% at 12 h and then leveled off, marking the entry of the drying process into a slow moisture-change stage. This transition in water status coincided with marked changes in non-volatile and volatile components. After 12 h, the decreases in most free amino acids and reducing sugars, as well as the consumption of linoleic acid, slowed markedly after 12 h; the contents of fruity compounds (ethyl isovalerate, β-ionone) and hexanal decreased by more than 50% within the first 12 h and then stabilized, whereas the lipid oxidation product (*E*)-2-nonenal began to increase continuously after 12 h. Correlation analysis revealed that free water and bound water were positively correlated with fruity compounds and (*E*)-2-nonenal, respectively, while linoleic acid was negatively correlated with (*E*)-2-nonenal. Principal component analysis and hierarchical cluster analysis further supported the identification of 12 h as a potential turning point for both water status and flavor transformation. Collectively, these findings suggest a coupling relationship between water status and flavor evolution during drying, providing a basis for optimizing vacuum freeze-drying processes to better preserve the characteristic aroma of mulberry.

## 1. Introduction

Mulberry (*Morus* spp.), a highly nutritious fruit, is rich in free acids, amino acids, vitamins, and functional bioactive compounds such as flavonoids, anthocyanins, and polysaccharides [1]. Due to its high perishability, only a limited quantity is marketed fresh, with the majority being processed to extend shelf life. Drying is one of the most prevalent preservation methods. Among various drying technologies, vacuum freeze-drying (VFD) stands out for its ability to effectively preserve the original shape, color, and nutritional quality of food while enabling rapid rehydration [2]. The low-temperature processing conditions of VFD minimize thermal degradation and lipid oxidation. However, the high vacuum and prolonged dehydration often result in the substantial loss of volatile flavor compounds, resulting in a muted or bland flavor profile [3].

In recent years, research on the flavor of dried mulberry has made certain progress. Yin et al. [4] compared five drying methods and identified 47 volatile compounds, revealing significant alterations in aldehydes, alcohols, and esters. Zhang et al. [5] further found that VFD mulberry retained the highest levels of anthocyanins, total flavonoids, and soluble sugars, with aldehydes being the most abundant volatiles, among which 2,6-nonadienal exhibited the highest odor activity value. Our previous work further identified six key aroma-active compounds in vacuum freeze dried mulberry [6]. However, these studies focused on endpoint differences or static component identification, failing to reveal the driving mechanisms of flavor evolution during drying.

As the most fundamental physicochemical change during drying, water status has been demonstrated to be closely related to flavor metabolism in food products. Li et al. [7] found in a study on grape freeze drying that the reduction in free water and weakly bound water was coupled with the migration of volatile compounds, and that water sublimation promoted the escape of flavor compounds from the matrix. Wang et al. [8] further confirmed in green pepper drying that the decrease in free water peak area monitored by low field nuclear magnetic resonance (LF-NMR) was positively correlated with the loss of 25 volatile compounds. Nevertheless, in the field of mulberry VFD, key questions such as how water status drives the stage specific changes in flavor compounds remain systematically unexplored. Furthermore, whether there exists a clear metabolic turning point at which flavor shifts from a rapid loss phase to a quasi-steady state is still unclear.

It should be noted that the critical transition point of flavor metabolism may be influenced by vacuum freeze-drying parameters such as pre-freezing temperature and vacuum pressure. Therefore, under fixed processing conditions, this study aims to elucidate the dynamic evolution of mulberry flavor during vacuum freeze-drying and to identify its critical metabolic transition point. The specific objectives are: (i) to monitor in real time the changes in water distribution, including bound water, immobile water, and free water, as well as non-volatile flavor precursors, including free amino acids, soluble sugars, organic acids, and fatty acids, and key aroma-active compounds over a 72 h drying process; (ii) to establish a correlation based framework linking water dynamics to flavor metabolite changes, thereby revealing the intrinsic mechanism by which water status drives flavor chemical transformations; and (iii) based on the above dynamic monitoring and correlation analysis, to identify the flavor transition point at which the flavor profile shifts from precursor degradation dominated to lipid oxidation derived aroma dominated, thus providing a theoretical basis for optimizing vacuum freeze drying processes to better preserve the characteristic fruity aroma of mulberry.

## 2. Materials and Methods

### 2.1. Materials and Reagents

The standard compounds used in this study were all commercially available products. 2-Methyl-3-heptanone (for GC use, purity 99.7%) was purchased from Sigma-Aldrich Co., Ltd. (Beijing, China). Sodium dihydrogen phosphate (analytical grade, purity 99%), n-hexane (purity 97%), methanol (purity 99.9%), acetonitrile (purity 99.9%), sodium hydroxide (analytical grade, purity 99%) were obtained from Beijing Merda Technology Co., Ltd. (Beijing, China). 9-Fluorenylmethyl chloroformate (FMOC), o-phthalaldehyde (OPA), amino acid mixture standard solution, and borate buffer solution were acquired from Agilent Corporation (Santa Clara, CA, USA). Reducing sugar standards, including glucose, fructose, sucrose, and maltose, were obtained from Yuanye Biotechnology Co., Ltd. (Shanghai, China). Organic acid standards such as citric acid, malic acid, tartaric acid, succinic acid, α-ketoglutaric acid, lactic acid, fumaric acid, and oxalic acid were also purchased from Yuanye Biotechnology Co., Ltd. (Shanghai, China). Thirty-seven fatty acid methyl ester standards (25 mg) were acquired from ANPEL-TRACE Standard Technical Services Co., Ltd. (Shanghai, China). Concentrated sulfuric acid (purity 95.0–98.0%) and hydrochloric acid (purity 36.0–38.0%) were procured from Sinopharm Chemical Reagent Co., Ltd. (Shanghai, China). Nitrogen (99.999%) was purchased from Beijing AP BAIF Gases Industry Co., Ltd. (Beijing, China).

### 2.2. Samples Preparation

FM: The selected mulberry variety was *Black Pearl*, purchased in 2023. The mulberries were washed and processed into pulp using a bench-scale juicer (Joyoung Co., Ltd., Qingdao, China). The FM was immediately sealed in aluminum foil vacuum bags, flash-frozen in liquid nitrogen, and stored in an ultra-low-temperature refrigerator at −80 °C for a maximum of 8 weeks.

VFDM: 20 g of fresh mulberry pulp was evenly distributed in a Φ 90 × 15 mm Petri dish and dried in an Alpha 1–4 LSC freeze dryer (Martin Christ, Osterode, Germany) at −55 °C (cold trap) and 22 Pa (absolute pressure) for 4, 8, 12, 16, 20, 24, 36, 48, and 72 h. After drying, the samples were immediately vacuum-sealed and stored at −80 °C until analysis. Prior to extraction, the dried samples were rehydrated to their original moisture content by adding distilled water based on the measured moisture loss rate at each drying time point, followed by vortex mixing to obtain a homogeneous reconstituted pulp.

Three independent batches (biological replicates) were prepared from the same homogenized pulp, and each batch was subjected to the freeze-drying process and subsequent analyses independently. All measurements were performed in triplicate analytical runs for each biological replicate. All concentrations are expressed on a fresh weight (FW) basis.

### 2.3. Moisture Determination

#### 2.3.1. Moisture Content

FM: The moisture content of the mulberry pulp was determined using the direct drying method [9].

VFDM: The moisture content of the VFDM samples was calculated based on the water loss rate during the freeze-drying process, according to Formula (1).

(1) $$\mathrm{Water}\;\mathrm{loss}\;\mathrm{rate}(\%)=100\;\times \;\left(\frac{M_{0}-M_{x}}{M_{0}}\right)$$

In the formula:

*M*_0_ is the initial mass of the sample, g;

*M*x is the sample mass after drying, g.

#### 2.3.2. Moisture Distribution

The water status of mulberries during drying was determined by a NIUSTEL-2 low-field nuclear magnetic resonance (LF-NMR) analyzer (Niumag, Shanghai, China) [10]. The spectrometer has a permanent magnet of 0.5 T and a radio frequency diameter of 60 mm. A standard copper sulfate solution was placed in the center of a radio frequency (RF) coil in the magnetic box. Using the free induction decay (FID) sequence, the center frequency of 23.04 MHz at 32 ± 0.01 °C was determined, and the proton resonance frequencies of 90° and 180° pulses were automatically searched, which were 17 and 35.04 μs, respectively. The decay relaxation time was TW = 5500 ms, the echo time TE was 0.5 ms, the return beam NECH was 1500, the sampling point TD was 150,042, and the number of repeated scans was NS = 32.

The mulberry samples were promptly transferred into 2 mL HPLC vials. After the samples equilibrated to room temperature, the vials were sealed with sealing film. Each vial was then placed into a specialized cylindrical glass tube for testing. The resonant center frequency was adjusted based on the FID signal, after which the transverse relaxation time (T_2_) was measured using the Carr–Purcell–Meiboom–Gill (CPMG) sequence. The acquired data were processed by inversion with the Simultaneous Iterative Reconstruction Technique (SIRT) to obtain the transverse relaxation spectrum of the sample. In the resulting spectrum, the horizontal and vertical axes represent the relaxation time and signal intensity, respectively.

### 2.4. Determination of Non-Volatile Compounds

#### 2.4.1. Determination of Free Amino Acids

The free amino acids in FM and VFDM were quantitatively analyzed by Agilent pre-column derivatization high-performance liquid chromatography (HPLC) [11]. Briefly, 5 g of fresh or freeze-dried rehydrated mulberry pulp was accurately weighed into a 50 mL centrifuge tube, mixed with 5 g of ultrapure water, and vortexed. The mixture was extracted in a water bath at 60 °C for 30 min, then centrifuged at 5000 rpm and 4 °C for 10 min using a SS-7 centrifuge (Hitachi, Tokyo, Japan). The supernatant was filtered through a 0.45 μm membrane filter and transferred into an HPLC vial for analysis. The chromatographic column was a Zorbax Eclipse-AAA column (4.6 mm × 150 mm, 5 μm; Agilent, Santa Clara, CA, USA). Mobile phase A was 40 mmol/L NaH_2_PO_4_ solution (pH 7.8), and mobile phase B was methanol–acetonitrile–water (45:45:10, *v*/*v*/*v*). The flow rate was 1 mL/min, column temperature was 40 °C, injection volume was 1 μL, and the detection wavelength was 338 nm using a diode array detector. A standard curve was established by gradient dilution of free amino acid standards with 0.1 mol/L HCl. Qualitative and quantitative analyses of free amino acids in the samples were performed based on retention time and peak area. Each sample was analyzed in triplicate.

#### 2.4.2. Determination of Soluble Sugars

Soluble sugars in FM and VFDM were determined using an Agilent 1260 high-performance liquid chromatography (HPLC) system (Agilent, Santa Clara, CA, USA) [12]. Briefly, 5 g of fresh or freeze-dried rehydrated mulberry pulp was accurately weighed into a centrifuge tube, mixed with 5 g of ultrapure water, vortexed, and then subjected to ultrasonic extraction for 30 min. The extract was centrifuged at 5000 rpm and 4 °C for 10 min. The supernatant was collected and diluted to 10 mL, then filtered through a 0.45 μm membrane filter and transferred into an HPLC vial for analysis. Chromatographic separation was performed on a Phenomenex Rezex ROA column (Phenomenex, Torrance, CA, USA) using 0.005 mol/L sulfuric acid as the mobile phase at a flow rate of 0.5 mL/min. The column temperature was maintained at 60 °C, the refractive index detector temperature was 40 °C, and the injection volume was 10 μL. Quantification was carried out using the external standard method. Standard solutions of glucose, fructose, sucrose, and maltose at different concentrations were prepared, and external calibration curves were constructed based on known concentrations and corresponding peak areas to calculate the soluble sugar contents in the samples. Each sample was analyzed in triplicate.

#### 2.4.3. Determination of Organic Acid Content

Organic acids in FM and VFDM were analyzed using an Agilent 1260 HPLC system equipped with a UV detector [13]. Briefly, 5 g of fresh or freeze-dried rehydrated mulberry pulp was accurately weighed into a centrifuge tube, mixed with 5 g of ultrapure water, vortexed, and then extracted by ultrasonication for 30 min. The extract was centrifuged at 5000 rpm and 4 °C for 10 min. The supernatant was collected, diluted to 10 mL, filtered through a 0.45 μm membrane filter, and transferred into an HPLC vial for analysis. Chromatographic separation was performed on a Phenomenex Rezex ROA column using 0.005 mol/L sulfuric acid as the mobile phase at a flow rate of 0.5 mL/min. The column temperature was maintained at 60 °C, the UV detection wavelength was 210 nm, and the injection volume was 10 μL. Quantification was carried out using the external standard method, and organic acid contents were calculated based on calibration curves. Each sample was analyzed in triplicate.

#### 2.4.4. Determination of Fatty Acid Content

Fatty acid content in the samples was determined using the external standard method [14]. Briefly, 4 mL of n-hexane was added to the sample for dissolution, followed by the addition of 200 μL of KOH-methanol solution (0.5 mol/L). The mixture was vigorously shaken for 30 s and then allowed to stand until clear. Then, 1 g of sodium hydrogen sulfate was added to neutralize the KOH. After the salt precipitated, the supernatant was collected for gas chromatography analysis. Chromatographic conditions: an Rt-2560 capillary column (100 m × 0.25 mm × 0.20 μm; Restek, Bellefonte, PA, USA) was used; the injector temperature was 270 °C and the detector temperature 280 °C. The temperature program was as follows: initial temperature 100 °C, ramped to 180 °C at 10 °C/min and held for 6 min, then increased to 200 °C at 1 °C/min and held for 20 min, and finally raised to 230 °C at 4 °C/min and held for 10.5 min. Nitrogen was used as the carrier gas with a split ratio of 100:1, and the injection volume was 1 μL. Quantification was performed using the external standard method. A series of fatty acid methyl ester mixed standard solutions at different concentrations were prepared to establish calibration curves, and the content of each fatty acid in the samples was calculated accordingly. Each sample was analyzed in triplicate.

### 2.5. Electronic-Nose Analysis

An electronic nose (PEN 3, Airsense, Schwerin, Germany) was used to discriminate the flavor difference between the FM samples and VFDM samples subjected to different drying times, according to the method of Song et al. [15]. A Luer Lock needle was inserted into the headspace of the sample vial, and filtered air was pumped into the sensor array at a constant flow rate of 120 mL/min. The sampling time was set to 240 s, and the sensor array cleaning time was set to 150 s. With sampling time as the horizontal axis and the response signal value (G/G_0_) as the vertical axis, ten curves in different colors were obtained, representing the response variations in the ten sensors over 240 s. The response values of the ten sensors are described as shown in Table 1.

### 2.6. Flavor Analysis

#### 2.6.1. SPME

The key aroma-active compounds were extracted from the samples using SPME, according to the method of Yang et al. [16]. 5 g of FM and VFDM samples were placed in a 20 mL headspace vial with 1 μL of 2-methyl-3-heptanone (0.816 μg/μL, internal standard), respectively. Afterward, the sample was extracted using an SPME sampler with DVB/CAR/PDMS fibers (50/30 μm, 2 cm; Supelco, Bellefonte, PA, USA) at 40 °C for 40 min. The fiber was then inserted into a GC injector and desorbed for 5 min at 230 °C. The experiment was repeated three times.

#### 2.6.2. GC-MS Analysis

A 7890B-5977A GC–MS (Agilent, Santa Clara, CA, USA) was used for the analysis of key aroma-active compounds in mulberry, following the method described by Yan et al. [17]. Chromatographic separations were performed using a DB-WAX polar column (60 m × 0.25 mm × 0.25 µm; Agilent, Santa Clara, CA, USA), with the inlet temperature maintained at 230 °C. The column conditions were set as follows: the initial column temperature was held at 40 °C for 5 min, then increased to 230 °C at a rate of 4 °C/min and held for another 5 min. The inlet temperature was 230 °C, and the injector was operated in split mode. The carrier gas was helium with a constant flow rate of 1 mL/min. EI mass spectra within 30–450 *m*/*z* were produced at 70 eV energy for the semi-quantitative analysis of volatiles in mulberry. SIM mode was adopted to accurately quantify aroma-active compounds in mulberry.

### 2.7. Data Analysis

All data are presented as mean ± standard deviation (SD) from three independent biological replicates (*n* = 3), with each replicate analyzed in triplicate. One-way analysis of variance (ANOVA) followed by Duncan‘s multiple range test was performed using IBM SPSS Statistics 26 to determine significant differences between groups, and a *p*-value < 0.05 was considered statistically significant. Bar charts, column graphs, radar charts, and PCA plots were generated using Origin 2024 software. Correlation heatmap was created using the Chiplot online platform (https://www.chiplot.com). Cluster analysis plot was generated using https://www.bioinformatics.com.cn.

## 3. Results

### 3.1. Moisture Distribution and Water State of VFDM

**Figure 1:**
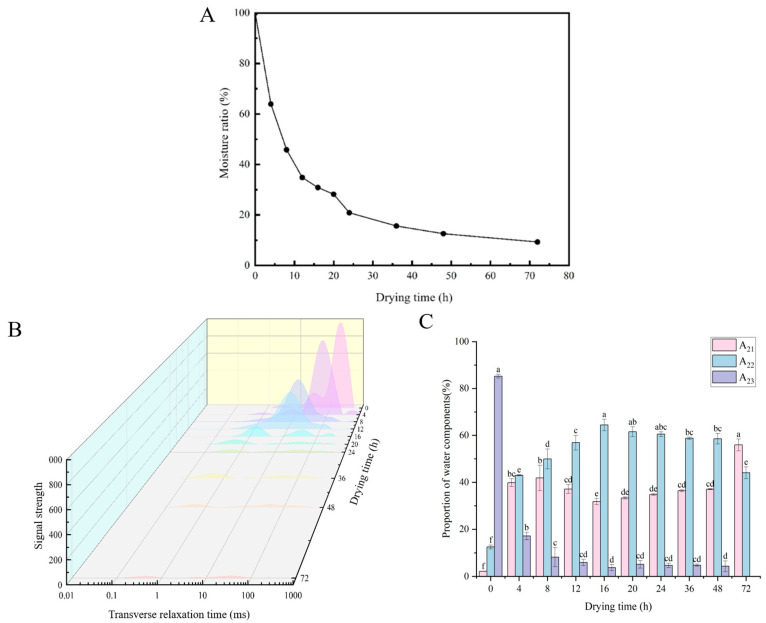
Dynamic changes in drying rate and water status during VFDM. (**A**) Drying curves (moisture ratio over drying time). (**B**) Transverse relaxation time spectra monitored by LF-NMR (colors indicate different drying times, 0–72 h, as shown in the embedded legend). (**C**) Relative proportions of bound water (A_21_), immobilized water (A_22_), and free water (A_23_) throughout the drying process. Different lowercase letters above the bars indicate significant differences (*p* < 0.05) for the same water component across different drying times.

The moisture content, distribution, and state of dried products significantly influence their flavor, texture, nutritional properties, functional characteristics, and shelf life [18]. The initial moisture content of FM was 85.344 ± 1.032%. As shown in Figure 1A, drying kinetics revealed a rapid initial moisture loss, with over 50% removed within the first 8 h, followed by a progressively slower rate in later stages. This pattern indicates that the process was predominantly governed by internal moisture diffusion (the falling rate period), consistent with the findings of Cui et al. [19].

The moisture distribution of VFDM was analyzed using LF-NMR. As shown in Figure 1B, the T_2_ relaxation spectrum revealed three distinct water states: T_21_ (0.01–10 ms), corresponding to water tightly bound to macromolecules; T_22_ (10–100 ms), representing immobile water; and T_23_ (100–1000 ms), characteristic of free water with the highest water activity [20]. The area under each peak is related to the content of the respective water component [21]. The changes in the relative content of each water component during the drying process are presented in Figure 1C, where A_21_, A_22_, and A_23_ represent bound water, immobilized water, and free water, respectively. In FM, A_23_ was found to account for 85.278%, with the highest T_23_ peak being observed, indicating high water mobility and loose binding with macromolecules. During the first 4–8 h of drying, a sharp decrease in A_23_ to 8.188% was detected, accompanied by a drastic attenuation of the T_23_ peak, while the proportions of A_21_ and A_22_ were increased [22]. At 12 h of drying, A_23_ had dropped to 5.96%, and thereafter it decreased only slightly and stabilized at 4–5%. Consistent with studies on the hot-air drying of American ginseng, the time point at which free water content reached equilibrium exactly coincided with the transition from the rapid drying stage to the falling-rate stage in the drying kinetics curve [23], indicating that 12 h is the critical turning point of the water status during the drying process. Between 16 and 48 h of drying, A_23_ was further decreased; the proportion of A_22_ was first increased to a peak of 64.419% at 16 h and then gradually declined, whereas A_21_ was steadily increased (to 37.079% at 48 h). By 72 h, free water was almost completely removed, A_22_ was increased to 55.941%, the T_23_ peak disappeared, and the T_21_ peak became dominant. This trend is consistent with the findings of Younas et al. [24] on VFD shiitake mushrooms, indicating that the sublimation dehydration process under low-temperature vacuum conditions is mild and helps preserve cell structure [25]. Studies on chicken drying have indicated that the transition of water status is often closely related to the subsequent formation of flavor-related metabolites [26]. Therefore, we further analyzed the dynamic changes in free amino acids, soluble sugars, fatty acids, and volatile components before and after drying.

### 3.2. Analysis of Free Amino Acids, Soluble Sugars, Organic Acids and Fatty Acids Contents in VFDM

#### 3.2.1. Free Amino Acids

A total of 14 free amino acids were identified in FM (Table 2), During VFD, these 14 free amino acids in mulberry were classified into four categories based on their taste characteristics: umami amino acids (glutamic acid, aspartic acid), sweet amino acids (alanine, glycine, serine, threonine), bitter amino acids (arginine, valine, leucine, isoleucine, phenylalanine, tyrosine, lysine), and sulfur-containing amino acids (cysteine) [27]. As drying time prolonged, all amino acids decreased significantly, with total amino acids decreasing by 38.2%, among which umami amino acids decreased by 46.7%, sweet amino acids by 35.0%, bitter amino acids by 42.5%, and cysteine by 43.1%. A similar trend of amino acid loss during freeze-drying has also been reported in other food matrices [28]. The loss of amino acids mainly occurred within the first 12 h, and the extent of decline slowed down markedly after 12 h. The substantial loss of amino acids directly weakened the umami and sweet taste of mulberry, but also greatly reduced the content of bitter amino acids. In addition, the consumption of cysteine [29] and the release of bound phenolic compounds [30] (e.g., guaiacol) provided precursors for the generation of sulfur off-flavors (garlic, sulfur-like) and smoky odors.

#### 3.2.2. Soluble Sugars

Soluble sugars are important precursors for the Maillard reaction and caramelization [31]. The soluble sugar content in mulberry accounts for approximately 10% of the fresh weight, mainly consisting of fructose and glucose in a ratio of nearly 1:1. As shown in Table 3, the fructose content (72.018 ± 0.020 mg/g) is slightly higher than that of glucose (69.230 ± 0.114 mg/g) in FM. During drying, both sugars showed a continuous downward trend, with a more pronounced reduction within the first 12 h, followed by a gradual leveling off thereafter, which is consistent with reports on freeze dried durian [32].

#### 3.2.3. Organic Acids

As shown in Table 4, the main organic acids in VFDM were citric acid (768.56–781.45 mg/100 g) and malic acid (469.64–495.94 mg/100 g), while tartaric acid and fumaric acid were present at very low levels (both <6 mg/100 g). During the entire drying process, the content of citric acid showed no significant change (*p* > 0.05). Malic acid content exhibited slight fluctuations but did not show a continuous decreasing or increasing trend with prolonged drying time, and the overall variation was relatively small. The contents of tartaric acid and fumaric acid showed no statistically significant differences at all time points (*p* > 0.05). In summary, VFD had no significant effect on the organic acid content of mulberry.

#### 3.2.4. Fatty Acids

The qualitative and quantitative results of fatty acids in VFDM are shown in Table 5. A total of five fatty acids were detected during the drying process: palmitic acid, stearic acid, oleic acid, linoleic acid, and linolenic acid. The content of each fatty acid gradually decreased with prolonged drying time, with significant differences between fresh and dried samples (*p* < 0.05) [33]. Among them, linoleic acid had the highest content, which was 23.016 ± 0.419 mg/100 g FW in fresh mulberry and decreased to 10.256 ± 1.271 mg/100 g FW after 72 h of drying. Linoleic acid decreased rapidly during the first 4 h of drying, followed by a slower decline between 4 and 12 h, and continued to decrease gradually thereafter until 72 h, which is consistent with the general pattern of substrate consumption during lipid oxidation. The other fatty acids were not detected in the early stage of drying, possibly because their contents were below the detection limit. Linoleic acid, oleic acid, and linolenic acid can all be converted into flavor compounds such as hexanal, hexanol, and nonanal through oxidative degradation [34].

### 3.3. E-Nose Analysis

**Figure 2:**
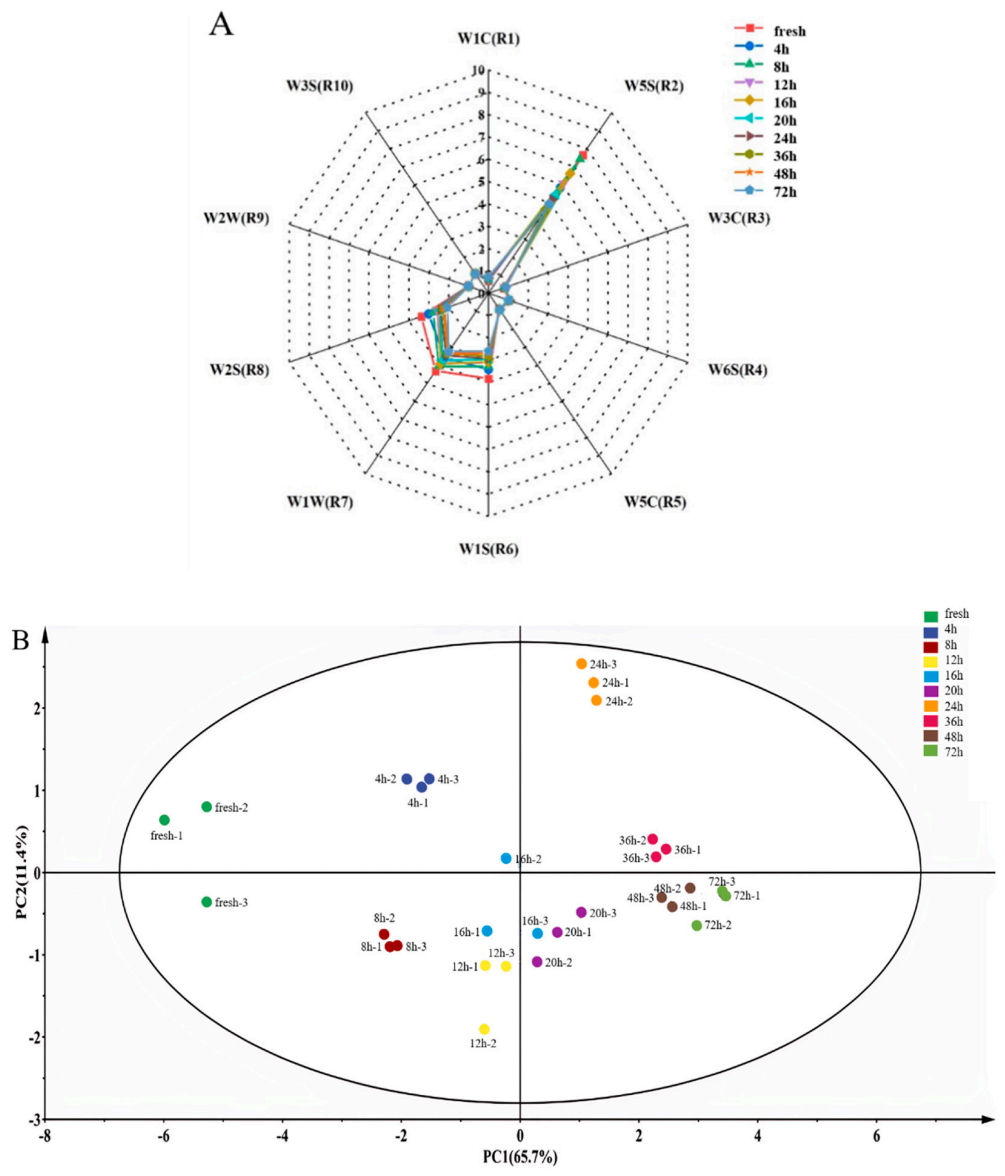
Aroma characteristics of VFDM pulp analyzed by electronic nose. (**A**) Radar chart of sensor response intensities. Sensors: W1C (aromatic compounds), W5S (nitrogen oxides), W3C (ammonia/aromatics), W6S (hydrogen), W5C (alkenes/aromatics), W1S (methyl compounds), W1W (sulfides), W2S (alcohols/aldehydes/ketones), W2W (aromatics/organic sulfides), and W3S (alkenes); (**B**) PCA score plot of samples at different drying stages.

The electronic nose identifies differences in volatile organic compounds through an odor sensor system and classifies different samples based on overall aroma information [35]. The volatile profiles of mulberry pulp at different drying stages were analyzed using an electronic nose, and the response intensities of the ten sensors are presented as a radar plot in Figure 2A. Among all sensors, W5S, W1S, W1W, and W2S exhibited the highest response values in FM samples, and their signals decreased progressively with increasing drying time, whereas the remaining six sensors showed no significant changes throughout the process. This suggests that VFD primarily led to the loss of volatile compounds such as sulfur-containing compounds, aldehydes/ketones, methyl compounds, and nitrogen oxides, likely due to their low boiling points or instability under high vacuum and prolonged drying conditions [36].

The principal component analysis (PCA) results based on electronic nose responses are shown in Figure 2B. The variance contribution rate of PC1 was 65.7%, and that of PC2 was 11.4%, with a cumulative variance contribution rate of 77.1%, indicating that the PCA model demonstrates the flavor distinctions among samples. Along the PC1 direction, samples from 0 to 12 h were distributed on the positive side, while samples from 16 to 72 h gradually shifted toward the negative side. Notably, the 12 h samples were located at the boundary between these two clusters, with highly concentrated sample points, suggesting that 12 h may represent a potential turning point where the flavor profile transitions from the rapid adjustment phase to the quasi-steady-state phase. The 16 h samples were more dispersed across both sides, further supporting the transitional nature of this period. The 12 h samples were exactly located at the boundary between the negative and positive sides of PC1, with the sample points being highly concentrated, indicating that this time point might be the potential turning point where the flavor profile shifts from the rapid adjustment phase to the quasi-steady-state phase.

### 3.4. Quantitative Analysis of Key Aroma-Active Compounds During VFD Process

Based on the previous identification of key aroma-active compounds in FM and VFDM, this study further monitored the content changes in six representative compounds (ethyl isovalerate, hexanal, (*E*)-2-nonenal, (*E,Z*)-2,6-nonadienal, β-ionone, and eugenol) during the drying process using SPME-GC-MS, in order to elucidate the flavor loss pattern of mulberry. As shown in Table 6, the contents of ethyl isovalerate, hexanal, and β-ionone decreased substantially during the first 12 h of drying, with losses exceeding 50%, which is consistent with the results of vacuum freeze-dried strawberry [37]. After 12 h, these three compounds showed an overall decreasing trend. In contrast, (*E*)-2-nonenal, (*E,Z*)-2,6-nonadienal, and eugenol slightly decreased during the initial stage, but increased after 12 h. Among them, (*E*)-2-nonenal was significantly increased at 12 h, while the absolute content of eugenol and (*E,Z*)-2,6-nonadienal was markedly increased at 16 h. Notably, the content of (*E,Z*)-2,6-nonadienal showed no significant change throughout the drying process (*p* > 0.05), and its dynamic change was different from that of (*E*)-2-nonenal. The increase of (*E*)-2-nonenal was associated with the substantial consumption of polyunsaturated fatty acids (especially linoleic acid) in the earlier stage, indicating that the lipid oxidation pathway was activated and became the dominant reaction generating aldehydes during the mid-to-late drying stages under low water activity [38]. The increase in eugenol content can be attributed to the loss of substantial amounts of free water, which caused the sublimation front to migrate inward, leading to the rupture of previously intact mulberry pulp cells and the release of bound eugenol [39].

### 3.5. Pearson Correlation Analysis Among Moisture, Free Amino Acids, Soluble Sugars, Fatty Acids and Key Aroma-Active Compounds

**Figure 3:**
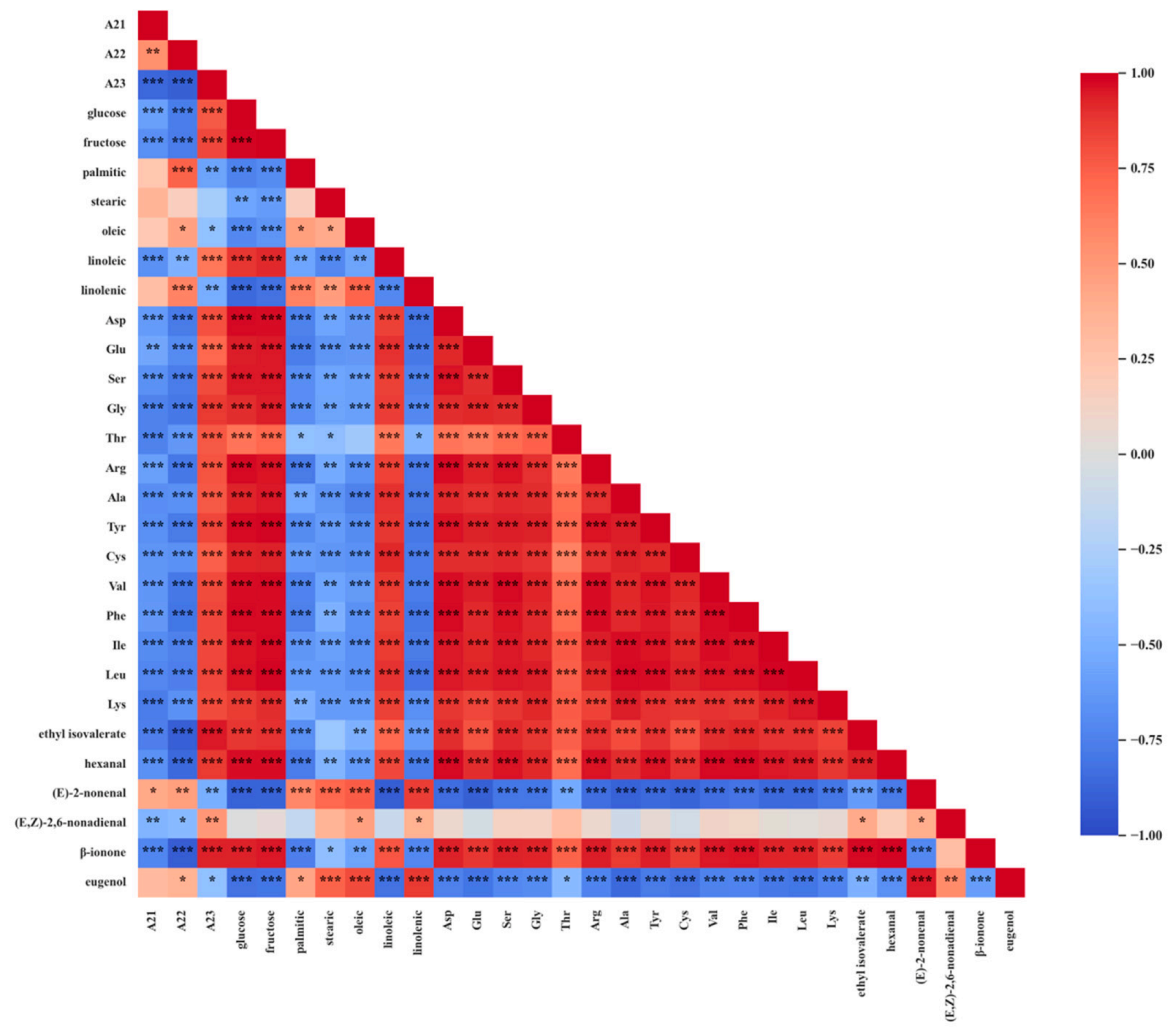
The Pearson correlation heatmaps of moisture, amino acids, sugars, fatty acids and aroma compounds. * *p* < 0.05; ** *p* < 0.01; *** *p* < 0.001.

To comprehensively elucidate the synergistic changes in metabolites during VFD, Pearson correlation analysis was performed on all measured indicators, including three water fractions (A_21_, A_22_, A_23_), two reducing sugars (glucose and fructose), five fatty acids, fourteen free amino acids, and six key aroma-active compounds, using data from three independent biological replicates at each of the ten drying time points (*n* = 30 per indicator) (Figure 3). The results revealed that A_23_ showed extremely significant positive correlations with fruity esters ethyl isovalerate (*r* = 0.96, *p* < 0.001) and β-ionone (*r* = 0.93, *p* < 0.001), but a moderate negative correlation with the lipid oxidation product (*E*)-2-nonenal (*r* = −0.48, *p* < 0.01). A_21_ was positively correlated with (*E*)-2-nonenal (*r* = 0.43, *p* < 0.05). These results indicated that free water, acting as a solvent and carrier of volatiles, drives the loss of fruity aromas [8], while bound water may provide a favorable microenvironment for lipid oxidation. Linoleic acid exhibited a strong negative correlation with (*E*)-2-nonenal (*r* = −0.92, *p* < 0.001) and a positive correlation with hexanal (*r* = 0.90, *p* < 0.001), confirming the lipid oxidation pathway as a major source of these aldehydes. Furthermore, several amino acids (e.g., glutamic acid, phenylalanine) also showed significant correlations with specific aroma compounds, indicating the participation of amino acid metabolism in flavor development. The above correlation results further support the intrinsic relationship between water status and metabolite changes during drying, providing a basis for determining the potential flavor transition point.

### 3.6. Identification and Validation of the Potential Flavor Transition Point During VFDM

#### 3.6.1. Identification of the Potential Flavor Transition Point

Integrating the dynamic changes in water status, non-volatile components, and key aroma-active compounds a consistent metabolic transition was clearly observed around 12 h of drying. In terms of water status, A_23_ decreased from an initial 85.3% to 5.96% at 12 h and thereafter showed only a slight decrease, stabilizing at 4–5%, indicating that free water was largely removed. For non-volatile components, the degradation rates of most free amino acids and reducing sugars slowed down markedly after 12 h, and the consumption of linoleic acid also gradually slowed after 12 h. Regarding volatile components, fruity esters (ethyl isovalerate, β-ionone) and hexanal lost more than 50% of their initial content within the first 12 h and then plateaued, whereas the lipid oxidation product (*E*)-2-nonenal began to increase continuously after 12 h. Although a few compounds (e.g., eugenol) showed significant accumulation only after 16 h, overall 12 h was the time point at which the trends of most indicators turned. Therefore, 12 h was identified as a potential turning point where the flavor profile shifted from a rapid adjustment phase to a quasi-steady-state phase.

#### 3.6.2. Validation by PCA Biplot

**Figure 4:**
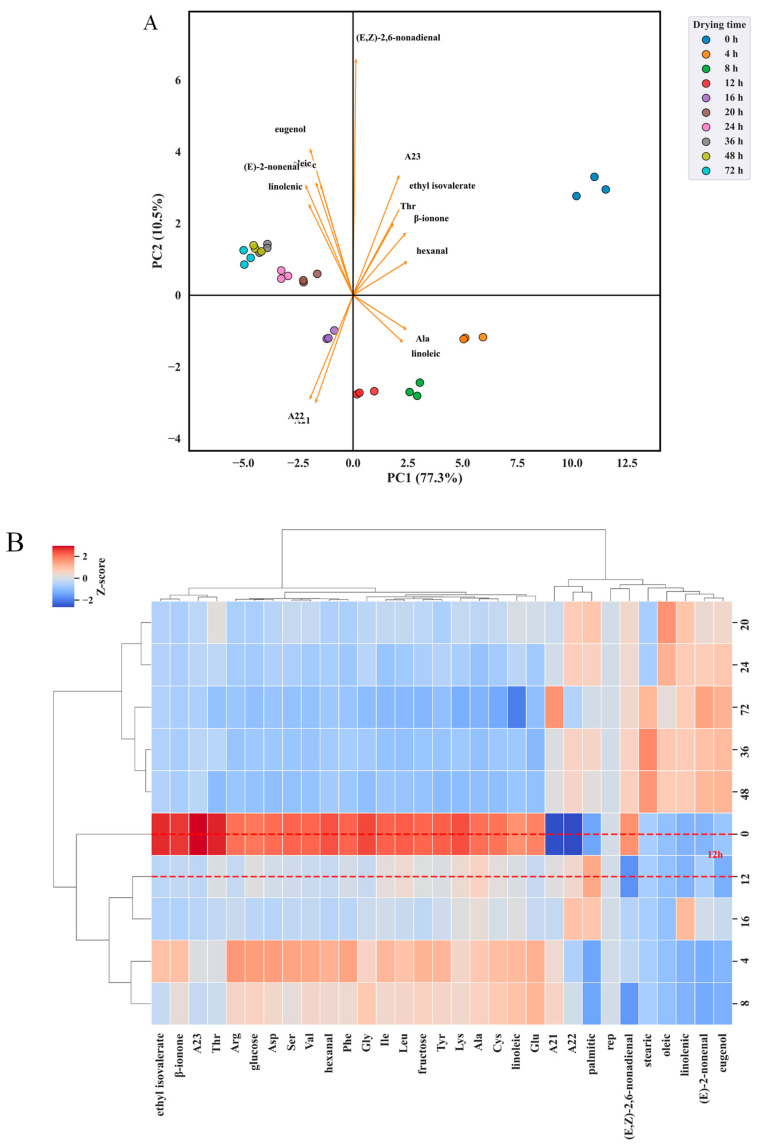
Multivariate statistical analysis of metabolic indicators during VFDM. (**A**) PCA biplot of samples at different drying times. (**B**) Hierarchical clustering heatmap of water status, non-volatile precursors, and volatile aroma compounds.

To visualise the overall trajectories of the indicators during drying, PCA was performed on the 15 variables with the highest contributions. As shown in Figure 4A, PC1 and PC2 explained 77.3% and 10.5% of the total variance, respectively, with a cumulative contribution of 87.8%, indicating that PCA effectively captured the overall characteristics of the drying process. In the PCA loading plot, each arrow corresponds to a variable, and the distance between the arrow and the sample points represents the degree of correlation, the closer the distance, the stronger the correlation. Samples from 0 h to 12 h were distributed on the positive side of PC1 (high-loading region), being mainly associated with A_23_, fruity compounds and some amino acids (e.g., threonine). In contrast, samples from 16 h to 72 h moved to the negative side of PC1 (low-loading region), being associated with lipid-oxidation products ((*E*)-2-nonenal, eugenol) and linoleic acid. This distribution clearly reveals the overall shift in flavor profiles from fruity to lipid-derived aromas during drying. The PCA trajectory shows that 12 h is the time point where the moving direction of the samples in PC space systematically changed.

#### 3.6.3. Validation by Hierarchical Cluster Analysis

Hierarchical cluster analysis was performed on all indicators (Figure 4B). The results showed good clustering of the changes and accumulation of different indicators during drying. Taking 0 h as a reference, the 4–8 h samples clustered together, and the 16–72 h samples formed another cluster, while 12 h lies on the boundary between the two clusters and is closer to the 16 h samples. The PCA and clustering results were highly consistent, supporting the identification of 12 h as a potential turning point in overall flavor transformation patterns.

## 4. Discussion

The rapid loss of free water within the first 12 h coincided with the substantial depletion of amino acids, reducing sugars, and fruity volatiles, suggesting that free water acts as the primary carrier for these compounds during sublimation. This is supported by the positive correlations between A_23_ and ethyl isovalerate (r = 0.96) and β-ionone (r = 0.93). The slowdown in precursor degradation after 12 h may be attributed to the limited mobility of reactants under low water activity. In contrast, the continuous increase of (*E*)-2-nonenal after 12 h implies that lipid oxidation became more active at this stage, possibly due to the concentration of unsaturated fatty acids. The negative correlation between linoleic acid and (*E*)-2-nonenal (r = –0.92) supports this pathway.

Several limitations should be acknowledged. First, the moisture content of VFDM samples at each time point was estimated from the water loss rate rather than directly measured, and water activity was not determined. Direct measurement of residual moisture and water activity would have provided more accurate water status characterization. Second, the correlation analysis was based on a limited number of samples (*n* = 10 time points), which may affect the statistical robustness of the results. Third, the limits of detection and quantification for the aroma compounds were not determined, potentially affecting the accuracy of trace-level quantification. Future work should directly measure residual moisture and water activity, validate the correlations with a larger sample set, and establish method detection limits. Additionally, model systems with controlled amino acid and sugar ratios could be used to further verify the precursor-product relationships suggested by the correlation analysis.

## 5. Conclusions

Water status played a central role in driving the flavor evolution of mulberry during vacuum freeze-drying. The drying process exhibited two distinct phases separated by 12 h, with the transition driven primarily by the depletion of free water. Before 12 h, free water decreased sharply from 85.3% to 5.96%, and this rapid water loss was accompanied by substantial degradation of amino acids (total decrease of 38.2%) and soluble sugars (glucose from 69.23 to 49.05 mg/g FW; fructose from 72.02 to 49.16 mg/g FW), as well as extensive loss of fruity volatiles; ethyl isovalerate, β-ionone, and hexanal all decreased by more than 50% within the first 12 h. These concurrent changes suggest that free water acted as the primary carrier for both water-soluble precursors and volatile compounds during the early sublimation stage. After 12 h, free water reached a plateau at approximately 5% and remained stable thereafter. In this low-moisture environment, the degradation of amino acids and soluble sugars slowed markedly, while lipid oxidation became more active, as indicated by the continuous decrease in linoleic acid (from 23.02 to 10.26 mg/100 g FW over 72 h) and the corresponding accumulation of its oxidation product (*E*)-2-nonenal (from 11.43 to 36.55 μg/kg). Correlation analysis further confirmed these associations: free water was positively correlated with fruity esters, while linoleic acid was negatively correlated with (*E*)-2-nonenal, suggesting that water status modulates the balance between volatile loss and lipid oxidation-derived formation. PCA and hierarchical cluster analysis consistently supported 12 h as the boundary of this flavor transformation. Collectively, these findings identify 12 h as a potential flavor transition point, where the flavor profile shifts from fruity-volatile dominance to lipid oxidation-derived aldehyde dominance. These results suggest that shortening the high-free-water stage via pre-dehydration or enhanced sublimation could better retain fruity aromas, while reducing oxygen exposure after 12 h may help suppress off-flavor formation, thus providing potential process optimization targets for industrial freeze-dried mulberry production.

## Figures and Tables

**Table 1:** Ten sensors for electronic nose.

No.	Sensor	Performance Description
1	W1C	Sensitive to aromatic compounds
2	W5S	Sensitive to nitrogen oxides, very responsive
3	W3C	Sensitive to ammonia and aromatic compounds
4	W6S	Selective to hydrides
5	W5C	Sensitive to alkenes and aromatic compounds
6	W1S	Sensitive to methyl groups
7	W1W	Sensitive to sulfides
8	W2S	Sensitive to alcohols, aldehydes, and ketones
9	W2W	Sensitive to aromatic compounds and organic sulfides
10	W3S	Mainly sensitive to alkenes

**Table 2:** Changes in amino acids content in VFDM pulp.

	Concentration (mg/100 g FW)									
	FM	VFD-4 h	VFD-8 h	VFD-12 h	VFD-16 h	VFD-20 h	VFD-24 h	VFD-36 h	VFD-48 h	VFD-72 h
Glu	55.08 ± 3.99 ^a^	46.11 ± 1.16 ^b^	44.98 ± 2.43 ^b^	25.39 ± 2.18 ^c^	25.26 ± 1.99 ^c^	28.40 ± 3.77 ^c^	18.40 ± 0.98 ^d^	13.30 ± 1.34 ^e^	15.18 ± 1.35 ^de^	14.76 ± 0.63 ^de^
Asp	100.90 ± 2.41 ^a^	94.49 ± 3.90 ^b^	82.84 ± 2.77 ^c^	77.28 ± 2.54 ^d^	73.15 ± 1.56 ^de^	73.15 ± 1.95 ^de^	71.70 ± 1.42 ^ef^	69.39 ± 2.44 ^efg^	66.99 ± 2.28 ^g^	68.38 ± 1.55 ^fg^
Ser	39.84 ± 1.27 ^a^	36.92 ± 1.45 ^b^	32.67 ± 1.15 ^c^	31.42 ± 0.92 ^cd^	30.68 ± 0.69 ^cde^	31.01 ± 1.26 ^cd^	29.63 ± 1.16 ^def^	28.82 ± 0.15 ^ef^	28.40 ± 1.06 ^f^	28.42 ± 1.12 ^f^
Arg	113.26 ± 3.35 ^a^	106.84 ± 3.92 ^b^	92.36 ± 2.09 ^c^	82.82 ± 1.64 ^d^	83.03 ± 3.37 ^d^	78.23 ± 2.71 ^de^	76.89 ± 1.91 ^e^	74.85 ± 2.66 ^e^	74.95 ± 2.37 ^e^	75.14 ± 3.07 ^e^
Gly	8.51 ± 0.30 ^a^	6.15 ± 0.24 ^b^	6.47 ± 0.39 ^b^	5.25 ± 0.16 ^c^	5.31 ± 0.16 ^c^	5.04 ± 0.48 ^cd^	4.96 ± 0.16 ^cd^	4.58 ± 0.47 ^de^	4.28 ± 0.15 ^e^	4.26 ± 0.30 ^e^
Ala	127.62 ± 3.69 ^a^	107.92 ± 5.62 ^b^	104.30 ± 2.37 ^bc^	103.75 ± 2.87 ^bc^	99.33 ± 1.96 ^c^	89.92 ± 3.92 ^d^	81.14 ± 1.33 ^e^	80.44 ± 1.46 ^e^	79.66 ± 2.39 ^e^	76.85 ± 1.47 ^e^
Cys	21.69 ± 1.21 ^a^	18.91 ± 0.79 ^b^	18.23 ± 0.36 ^bc^	16.75 ± 0.44 ^cd^	16.14 ± 0.33 ^d^	15.42 ± 0.57 ^de^	14.20 ± 0.68 ^ef^	13.94 ± 0.59 ^efg^	13.39 ± 1.19 ^fg^	12.33 ± 2.26 ^g^
Tyr	19.59 ± 0.52 ^a^	17.16 ± 0.36 ^b^	15.82 ± 0.23 ^c^	14.84 ± 0.42 ^d^	14.47 ± 0.30 ^d^	13.43 ± 0.46 ^e^	13.59 ± 0.27 ^e^	12.82 ± 0.18 ^f^	12.41 ± 0.19 ^f^	12.58 ± 0.21 ^f^
Thr	42.50 ± 1.84 ^a^	35.18 ± 1.85 ^b^	34.56 ± 2.20 ^b^	35.19 ± 1.36 ^b^	34.12 ± 2.45 ^b^	35.47 ± 2.00 ^b^	34.06 ± 2.37 ^b^	33.82 ± 4.02 ^b^	32.09 ± 1.42 ^b^	32.42 ± 2.05 ^b^
Val	78.28 ± 1.76 ^a^	70.97 ± 2.10 ^b^	63.13 ± 1.54 ^c^	57.65 ± 1.75 ^d^	56.59 ± 0.77 ^d^	56.77 ± 2.34 ^d^	52.81 ± 1.94 ^e^	51.72 ± 2.49 ^e^	51.19 ± 2.14 ^e^	51.00 ± 1.77 ^e^
Phe	48.26 ± 1.66 ^a^	44.64 ± 1.26 ^b^	39.77 ± 0.66 ^c^	37.88 ± 0.92 ^d^	36.25 ± 0.60 ^de^	35.26 ± 1.07 ^ef^	34.33 ± 0.76 ^f^	34.93 ± 0.30 ^ef^	34.40 ± 1.30 ^f^	33.99 ± 0.32 ^f^
Lys	19.46 ± 2.20 ^a^	13.55 ± 1.37 ^b^	13.43 ± 1.23 ^b^	13.27 ± 1.02 ^b^	12.65 ± 0.70 ^b^	11.47 ± 1.27 ^bc^	10.27 ± 0.36 ^cd^	9.89 ± 0.84 ^cd^	9.06 ± 0.77 ^d^	8.27 ± 0.96 ^d^
Ile	43.74 ± 1.21 ^a^	37.61 ± 1.18 ^b^	33.53 ± 1.40 ^c^	32.65 ± 0.79 ^cd^	30.83 ± 1.33 ^de^	29.75 ± 1.63 ^e^	26.51 ± 0.72 ^f^	26.34 ± 2.04 ^f^	26.73 ± 1.29 ^f^	25.34 ± 0.90 ^f^
Leu	38.27 ± 1.14 ^a^	32.52 ± 0.62 ^b^	30.77 ± 0.73 ^c^	30.31 ± 0.46 ^d^	27.84 ± 0.73 ^e^	27.55 ± 0.49 ^e^	25.55 ± 0.60 ^f^	24.68 ± 0.53 ^fg^	24.95 ± 0.45 ^fg^	24.08 ± 0.60 ^g^
TEAA	270.51 ± 8.83 ^a^	234.48 ± 3.25 ^b^	215.19 ± 5.54 ^c^	206.96 ± 5.11 ^cd^	198.27 ± 4.00 ^de^	196.27 ± 8.22 ^e^	183.53 ± 4.85 ^f^	181.38 ± 3.74 ^f^	178.42 ± 2.07 ^f^	175.10 ± 3.89 ^f^
TAA	756.98 ± 26.55 ^a^	668.98 ± 25.81 ^b^	612.85 ± 19.54 ^c^	564.45 ± 17.47 ^d^	545.64 ± 16.95 ^e^	530.88 ± 23.93 ^e^	494.05 ± 14.67 ^f^	479.50 ± 19.49 ^fg^	473.67 ± 18.36 ^g^	467.82 ± 17.19 ^g^

Values are expressed as mean ± standard deviation of triplicate analysis. ^a–g^ Values bearing different superscript lowercase letters within the same column are significantly different (*p* < 0.05).

**Table 3:** Changes in soluble sugar content in VFDM pulp.

Drying Time	Concentration (mg/g FW)	
	Glucose	Fructose
0 h	69.23 ± 0.11 ^a^	72.02 ± 0.02 ^a^
4 h	66.08 ± 0.33 ^b^	64.97 ± 0.74 ^b^
8 h	59.58 ± 0.28 ^c^	60.90 ± 0.04 ^c^
12 h	56.86 ± 1.00 ^d^	57.92 ± 0.12 ^d^
16 h	53.10 ± 0.25 ^e^	54.63 ± 0.99 ^e^
20 h	51.64 ± 0.37 ^f^	54.33 ± 0.30 ^e^
24 h	50.61 ± 0.11 ^g^	52.71 ± 0.46 ^f^
36 h	50.90 ± 0.45 ^g^	51.22 ± 0.94 ^g^
48 h	49.43 ± 0.42 ^h^	50.59 ± 0.66 ^g^
72 h	49.05 ± 0.28 ^h^	49.16 ± 0.56 ^h^

Values are expressed as mean ± standard deviation of triplicate analysis. ^a–h^ Values bearing different superscript lowercase letters within the same column are significantly different (*p* < 0.05).

**Table 4:** Changes in organic acids content in VFDM pulp.

Drying Time	Concentration (mg/100 g FW)			
	Citric Acid	Malic Acid	Tartaric Acid	Fumaric Acid
0 h	781.45 ± 6.55 ^a^	492.82 ± 7.49 ^a^	5.87 ± 0.47 ^a^	2.07 ± 0.13 ^a^
4 h	778.10 ± 4.36 ^a^	494.82 ± 8.70 ^b^	5.56 ± 0.73 ^a^	2.08 ± 0.32 ^a^
8 h	776.58 ± 32.93 ^a^	485.27 ± 19.31 ^ab^	5.50 ± 0.63 ^a^	2.05 ± 0.40 ^a^
12 h	778.48 ± 17.87 ^a^	485.16 ± 11.17 ^ab^	5.20 ± 0.31 ^a^	2.05 ± 0.19 ^a^
16 h	773.50 ± 12.46 ^a^	471.62 ± 2.92 ^b^	5.30 ± 0.58 ^a^	2.03 ± 0.33 ^a^
20 h	771.68 ± 9.86 ^a^	469.64 ± 6.99 ^b^	5.23 ± 0.44 ^a^	2.04 ± 0.08 ^a^
24 h	770.06 ± 2.24 ^a^	494.87 ± 1.72 ^a^	5.36 ± 0.07 ^a^	2.02 ± 0.19 ^a^
36 h	768.53 ± 0.72 ^a^	484.71 ± 0.35 ^ab^	5.26 ± 0.58 ^a^	2.03 ± 0.13 ^a^
48 h	770.27 ± 11.56 ^a^	495.94 ± 0.53 ^a^	5.20 ± 0.97 ^a^	2.01 ± 0.04 ^a^
72 h	768.56 ± 6.92 ^a^	488.14 ± 4.32 ^a^	5.18 ± 0.19 ^a^	1.95 ± 0.06 ^a^

Values are expressed as mean ± standard deviation of triplicate analysis. ^a,b^ Values bearing different superscript lowercase letters within the same column are significantly different (*p* < 0.05).

**Table 5:** Changes in fatty acids content in VFDM pulp.

Drying Time	Concentration (mg/100 g FW)				
	Palmitic Acid	Stearic Acid	Oleic Acid	Linoleic Acid	Linolenic Acid
0 h	ND	ND	ND	23.02 ± 0.42 ^a^	ND
4 h	ND	ND	ND	20.87 ± 0.13 ^b^	ND
8 h	ND	ND	ND	20.37 ± 0.57 ^b^	ND
12 h	4.61 ± 0.15 ^a^	ND	ND	17.43 ± 0.71 ^c^	ND
16 h	3.79 ± 0.09 ^b^	ND	ND	17.74 ± 0.54 ^c^	0.97 ± 0.05 ^a^
20 h	3.77 ± 0.11 ^b^	ND	2.43 ± 0.03 ^a^	17.26 ± 0.59 ^c^	0.86 ± 0.03 ^b^
24 h	3.21 ± 0.19 ^c^	ND	2.04 ± 0.01 ^b^	15.81 ± 0.85 ^d^	0.83 ± 0.02 ^b^
36 h	3.35 ± 0.12 ^c^	1.35 ± 0.02 ^a^	1.63 ± 0.04 ^c^	14.51 ± 0.84 ^e^	0.81 ± 0.01 ^b^
48 h	2.72 ± 0.21 ^d^	1.26 ± 0.02 ^b^	1.57 ± 0.09 ^c^	14.33 ± 0.51 ^e^	0.81 ± 0.01 ^b^
72 h	2.33 ± 0.10 ^e^	0.98 ± 0.03 ^c^	1.12 ± 0.04 ^d^	10.26 ± 1.27 ^f^	0.81 ± 0.00 ^b^

Values are expressed as mean ± standard deviation of triplicate analysis. ^a–f^ Values bearing different superscript lowercase letters within the same column are significantly different (*p* < 0.05). ND indicates not detected.

**Table 6:** Changes in key aroma-active compounds in VFDM pulp.

Drying Time	Concentration (μg/kg)					
	Ethyl Isovalerate	Hexanal	(*E*)-2-Nonenal	(*E,Z*)-2,6-Nonadienal	β-Ionone	Eugenol
0 h	5.23 ± 0.60 ^a^	57.08 ± 4.04 ^a^	11.43 ± 0.83 ^g^	2.16 ± 0.09 ^a^	1.91 ± 0.04 ^a^	2.70 ± 0.27 ^f^
4 h	3.40 ± 0.16 ^b^	43.06 ± 3.97 ^b^	10.28 ± 2.27 ^g^	2.00 ± 0.07 ^bc^	1.03 ± 0.09 ^b^	2.15 ± 0.01 ^g^
8 h	2.23 ± 0.12 ^c^	32.71 ± 1.88 ^c^	9.61 ± 0.62 ^g^	1.90 ± 0.04 ^c^	0.68 ± 0.02 ^c^	2.26 ± 0.11 ^g^
12 h	2.09 ± 0.10 ^c^	25.16 ± 1.23 ^d^	16.68 ± 1.73 ^f^	1.89 ± 0.08 ^c^	0.38 ± 0.02 ^d^	2.13 ± 0.01 ^g^
16 h	1.95 ± 0.06 ^c^	22.38 ± 0.48 ^de^	22.31 ± 2.46 ^e^	1.99 ± 0.08 ^bc^	0.22 ± 0.01 ^e^	3.52 ± 0.08 ^e^
20 h	1.93 ± 0.08 ^c^	21.14 ± 1.01 ^e^	26.16 ± 1.29 ^d^	2.06 ± 0.09 ^ab^	0.26 ± 0.01 ^e^	4.35 ± 0.03 ^d^
24 h	1.94 ± 0.06 ^c^	20.18 ± 1.29 ^e^	29.47 ± 1.39 ^c^	2.06 ± 0.04 ^ab^	0.22 ± 0.00 ^e^	4.83 ± 0.06 ^c^
36 h	1.90 ± 0.21 ^c^	19.31 ± 0.91 ^e^	31.53 ± 3.10 ^bc^	2.07 ± 0.070 ^ab^	0.21 ± 0.01 ^e^	5.10 ± 0.01 ^b^
48 h	1.93 ± 0.08 ^c^	18.85 ± 1.87 ^e^	33.48 ± 2.13 ^ab^	2.08 ± 0.04 ^ab^	0.21 ± 0.00 ^e^	5.25 ± 0.06 ^ab^
72 h	1.90 ± 0.03 ^c^	18.56 ± 0.53 ^e^	36.55 ± 0.81 ^a^	2.06 ± 0.11 ^ab^	0.21 ± 0.00 ^e^	5.35 ± 0.05 ^a^

Values are expressed as mean ± standard deviation of triplicate analysis. ^a–g^ Values bearing different superscript lowercase letters within the same column are significantly different (*p* < 0.05).

## Data Availability

The original contributions presented in this study are included in the article. Further inquiries can be directed to the corresponding author.
